# Does the Kis-Balaton Water Protection System (KBWPS) Effectively Safeguard Lake Balaton from Toxic Cyanobacterial Blooms?

**DOI:** 10.3390/microorganisms9050960

**Published:** 2021-04-29

**Authors:** Zoran Marinović, Nada Tokodi, Damjana Drobac Backović, Ilija Šćekić, Nevena Kitanović, Snežana B. Simić, Nevena B. Đorđević, Árpád Ferincz, Ádám Staszny, Tamara Dulić, Jussi Meriluoto, Béla Urbányi, Jelena Lujić, Zorica Svirčev

**Affiliations:** 1 Department of Biology and Ecology, Faculty of Sciences, University of Novi Sad, 21000 Novi Sad, Serbia; nada.tokodi@dbe.uns.ac.rs (N.T.); damjana.drobac@dbe.uns.ac.rs (D.D.B.); Jussi.Meriluoto@abo.fi (J.M.); zorica.svircev@dbe.uns.ac.rs (Z.S.); 2 Department of Aquaculture, Institute of Aquaculture and Environmental Safety, Hungarian University of Agriculture and Life Sciences, 2100 Gödöllő, Hungary; ilijas92@gmail.com (I.Š.); nevena.n.kitanovic@gmail.com (N.K.); Urbanyi.Bela@uni-mate.hu (B.U.); 3 Laboratory of Metabolomics, Faculty of Biochemistry, Biophysics and Biotechnology, Jagiellonian University, 30387 Krakow, Poland; 4 Department of Biology and Ecology, Faculty of Science, University of Kragujevac, 34000 Kragujevac, Serbia; snezana.simic@pmf.kg.ac.rs (S.B.S.); nevena.djordjevic@pmf.kg.ac.rs (N.B.Đ.); 5 Department of Freshwater Fish Ecology, Institute of Aquaculture and Environmental Safety, Hungarian University of Agriculture and Life Sciences, 2100 Gödöllő, Hungary; Ferincz.Arpad@uni-mate.hu (Á.F.); Staszny.Adam@uni-mate.hu (Á.S.); 6 Faculty of Science and Engineering, Biochemistry, Åbo Akademi University, 20520 Turku, Finland; waterlifea@gmail.com; 7 Center for Reproductive Genomics, Department of Biomedical Sciences, Cornell University, Ithaca, NY 14850, USA; jelenalujic@cornell.edu

**Keywords:** cyanobacteria, cyanotoxins, microcystin, Hungary, histopathology

## Abstract

Lake Balaton is the largest shallow lake in Central Europe. Its water quality is affected by its biggest inflow, the Zala River. During late 20th century, a wetland area named the Kis-Balaton Water Protection System (KBWPS) was constructed in the hopes that it would act as a filter zone and thus ameliorate the water quality of Lake Balaton. The aim of the present study was to test whether the KBWPS effectively safeguards Lake Balaton against toxic cyanobacterial blooms. During April, May, July and September 2018, severe cyanobacterial blooming was observed in the KBWPS with numbers reaching up to 13 million cells/mL at the peak of the bloom (July 2018). MC- and STX-coding genes were detected in the cyanobacterial biomass. Five out of nine tested microcystin congeners were detected at the peak of the bloom with the concentrations of MC-LR reaching 1.29 µg/L; however, accumulation of MCs was not detected in fish tissues. Histopathological analyses displayed severe hepatopancreas, kidney and gill alterations in fish obtained throughout the investigated period. In Lake Balaton, on the other hand, cyanobacterial numbers were much lower; more than 400-fold fewer cells/mL were detected during June 2018 and cyanotoxins were not detected in the water. Hepatic, kidney and gill tissue displayed few alterations and resembled the structure of control fish. We can conclude that the KBWPS acts as a significant buffering zone, thus protecting the water quality of Lake Balaton. However, as MC- and STX-coding genes in the cyanobacterial biomass were detected at both sites, regular monitoring of this valuable ecosystem for the presence of cyanobacteria and cyanotoxins is of paramount importance.

## 1. Introduction

Lake Balaton is the largest Central European lake, located in Western Hungary [1,2]. With its large size, the lake has 51 inflows, with 20 of them being permanent [3]. The largest is the Zala River which accounts for approximately half of the water inflow. Due to this inflow capacity, and the fact that approximately 35–40% of the lake’s nutrient load originates from this river [2], it has a significant effect on the lake’s water quality. Zala enters the lake through a large wetland area named the Kis-Balaton Wetlands (KBW) which ‘filters’ sediments and nutrients which would otherwise enter the lake [1]. In the 18th and 19th century, this area was a large open water body surrounded by extensive wetland vegetation, including a wetland forest which concealed the Zala River flowing into Lake Balaton. This provided hydraulic resistance for Zala’s water and enabled eutrophication to occur before the water entered Balaton. However, the filtering capacity of the KBW was greatly compromised in the 19th and 20th century when the Sió Canal (the lake’s only outflow) was first widened to lower the water level of Lake Balaton in order to protect the newly built Budapest-Rijeka railway from flooding [2]. This caused a significant drainage of the KBW as well. KBW marshland was further drained by constructing large numbers of drainage canals in order to create additional agricultural land. With the KBW severely compromised, and with an increased utilization of phosphorus-rich artificial fertilizers in agricultural practice and increased sewage effluents from rapidly developing towns, as well as discharges from animal farms, an intense eutrophication of Lake Balaton started to occur in 1930s and 1940s [1,4]. The lake suffered its highest eutrophication in 1960s and 1970s when intensive cyanobacterial blooms started to occur [1,4]. At this time, it became evident that measures for alleviating this pressure on Balaton needed to be conducted, otherwise its developing economic potential as a tourist destination would be greatly threatened.

In order to decrease cyanobacterial blooms and further deterioration of water quality, inflow of organic nutrients needed to be reduced [2]. As wetlands are known to retain nutrients through several physical, chemical and biological factors such as physical sedimentation, adsorption of nutrients by the sediment, enhanced denitrification and uptake of nutrients by macrophytes or algae [5], re-flooding of the KBW (which would once again act as a ‘filter’ for Balaton) was planned under the framework of the Kis-Balaton Water Protection System (KBWPS). Re-flooding was planned in two phases: the first step would be the flooding of the Lower Zala Valley, while the second would be the flooding of the Kis-Balaton Basin [1]. Phase 1 was conducted in 1985 by a five-step flooding intervention and creation of an open waterspace (Hídvégi Pond) with a water retention time of 30 days [2,4,6,7]. Phase 2 was initiated in 1992 when a part of the 57 km^2^ Fenéki Pond was flooded. Only 16 km^2^ were flooded (with hydraulic retention time of 90 days) and they represent a typical wetland covered with macrophytes, primarily reeds. After Phase 1, 60% of the suspended solids coming from Zala River were retained in the first ponds, while after Phase 2, an additional 75% of suspended solids coming from Hídvégi Pond was retained [4].

Cyanobacterial blooms characterized by an overgrowth of cyanobacteria have become a great global problem, and their occurrence is continuously increasing [8,9,10]. These events cause major problems for water quality, for example, blooms can cause oxygen depletion in the ecosystem, sometimes even leading to hypoxia which can have severe negative consequences for the organisms living in such ecosystems [11]; increased turbidity that can smother aquatic vegetation [8]; and they can even produce a distinct taste and odor which can interfere with the function and use of the reservoir affected. Cyanobacterial blooms can pose a serious threat to public health when associated with the production of toxic compounds known as cyanotoxins [9,10,12]. Cyanotoxins are very diverse in their chemical structure and functional properties; based on their structure, they can be cyclic peptides, alkaloids, lipopeptides, non-protein amino acids and lipoglycans, while based on their functional properties they can be hepatotoxins, neurotoxins, cytotoxins and dermatotoxins [13,14]. Due to the negative effects cyanobacteria and their toxins can have on the environment and public health, regular monitoring of ecosystems susceptible to blooming is of great importance.

Lake Balaton has been extensively studied with respect to its nutrient status and cyanobacterial blooms since the 1960s [1,4,6,15]. However, the toxicity of these blooms and their effects on biota have not been considered to date. The aim of this study was to verify whether the KBWPS indeed acts as a ‘filter’ for Lake Balaton and ‘protects’ it from cyanobacterial blooms by: (1) assessing the overall water quality parameters for both ecosystems; (2) determining the qualitative and quantitative structure of the cyanobacterial communities; (3) testing for the presence of cyanotoxin-coding genes within the collected biomass; (4) analyzing the cyanotoxin presence in the water and cyanobacterial biomass; (5) assessing potential cyanotoxin accumulation in fish tissues; (6) determining possible cyanotoxin effects on fish tissues through histological analyses of various organs of fish from both the KBWPS and Lake Balaton.

## 2. Materials and Methods

Sampling conducted in the study was approved by the National Office of Environment, Nature and Water Conservation: license OKTF-KP/8294-14/2016, for research in the protected areas of the Balaton catchment and by the Hungarian Ministry of Agriculture: license HHgF/122-1/2018, for collecting and sampling fish from natural populations.

### 2.1. Study Area and Sampling Sites

**Figure 1:**
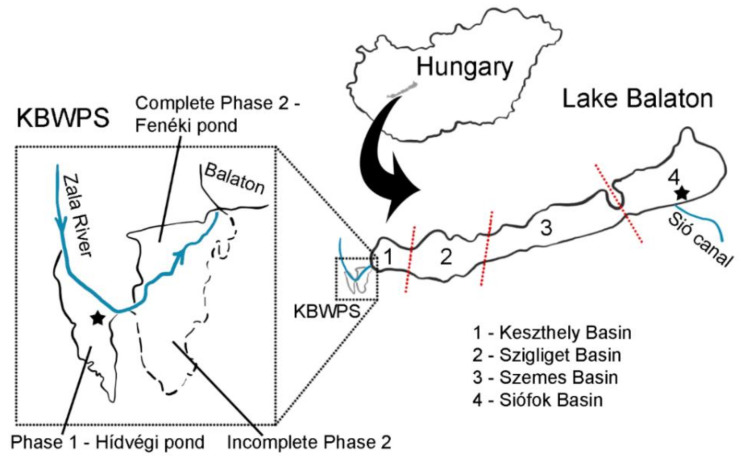
Map of Hungary and graphical depictions of the Kis-Balaton Water Protection System (KBWPS) and Lake Balaton. Sampling sites are labeled with an asterisk. Figure is based on those in Kovács et al. [2] and Hatvani et al. [16] with permission from the publisher.

Lake Balaton is the largest Central European shallow lake with a surface area of over 590 km^2^, a catchment area of over 5100 km^2^ and a mean depth of 3.25 m [1,2]. The lake is divided into four basins based on historical aspects and a clear trophic gradient: Basin 1—Keszthely Bay; Basin 2—Szigliget Basin; Basin 3—Szemes Basin; Basin 4—Siófok Basin (Figure 1). The western-most basin (Basin 1) is the most eutrophic, while other basins become less eutrophic along the gradient towards the east. The largest inflow of the lake, the Zala River, enters Lake Balaton at its western end, and it inflows into the Keszthey Bay. As mentioned before, the KBWPS is located at the mouth of Zala River into the lake. It consists of two main parts/phases: (1) Phase 1 which represents an open waterspace named Hídvégi Pond, with a surface area of 18 km^2^, mean depth of 1.1 m, and (2) Phase 2 named Fenéki Pond which is a typical wetland covered with macrophytes with a surface area of 16 km^2^. In spite of having many inflows, Lake Balaton has only one outflow—the Sió Canal located on the southeastern bank in Basin 4.

Sampling in the KBWPS was conducted during four months (April, May, July and September 2018) at the Hídvégi Pond (46°38.1′ N; 17°9.8′ E). During June of the same year, sampling was conducted in Lake Balaton in the Siófok Basin (Basin 4) near the Sió Canal (46°55′ N; 18°3.1′ E).

### 2.2. Sampling of Water and Fish

Water samples for cyanobacterial community analyses and cyanotoxin analyses were collected similarly as described in Tokodi et al. [17]. In short, 12 L of water for qualitative and quantitative analyses of cyanobacterial communities were collected by sweeping with a plankton net (netframe 25 cm, mesh size 23 µm) at a depth of 0.3 m. Samples were immediately fixed in 10% natural buffered formalin. Two liters of water were also collected for cyanotoxin analyses at each sampling time.

Sampling of fish was conducted on the same dates and at the same locations as water sampling (Table 1). Fish sampling was conducted with a standard electrofishing device. All fish were sacrificed by a blow to the head and tissues were immediately sampled on-site. An incision was made near the anal opening, and a cut was made along the belly of the fish by cutting the skin and underlying muscle, thus exposing all internal organs. Samples of hepatopancreas, kidney, intestines, spleen, gonads and gills were collected and immediately frozen for the qualitative and quantitative analyses of cyanotoxin presence. Hepatopancreas, kidneys, gills, spleen, gonads and muscles were also sampled in 10% neutral buffered formalin for histological analyses. Additionally, three common carp individuals from the regular broodstock of the Department of Aquaculture, Hungarian University of Agriculture and Life Sciences, Hungary, were sacrificed and used as a control. Fish were maintained at 24 ± 1 °C in a 12 h light/12 h dark cycle and were fed twice a day with commercial feed.

### 2.3. Analyses of Water Quality Parameters

Water quality parameters were measured on-site. Temperature, conductivity, pH and O_2_ concentrations were measured using a Hanna HI 98194 (Woonsocket, RI, USA) multiparametric device, while NH_4_-N, NO_3_-N, NO_2_-N, PO_4_-P concentrations were measured using a Machery-Nagel PF-12 (Düren, Germany) Plus spectrophotometer.

### 2.4. Qualitative and Quantitative Analyses of Cyanobacterial Communities

Taxonomic identification of cyanobacteria was conducted similarly to Tokodi et al. [16] and according to different taxonomic keys [18,19,20]. Identification was conducted under a Motic BA310 (Wetzlar, Germany) light microscope using a Bresser (9MP) digital camera and Micro Cam Lab software (Bonneuil, France). Quantitative analyses were conducted under a Motic AE2000 (Wetzlar, Germany) inverted microscope according to the Utermöhl method [21], where the phytoplankton was sedimented, and cyanobacteria were quantified in a counting chamber under different magnifications depending on cell size. Numbers of cyanobacteria are expressed as numbers of cells per mL.

### 2.5. Determination of Cyanotoxin-Coding Genes

Water samples (100–250 mL depending on the bloom intensity) were filtered through 2–3 µm filters, and the biomass was freeze-dried and subsequently used for genetic analysis. The methodology used for the qualitative determination of the presence of cyanotoxin-coding genes was described previously [16]. In short, approximately 10 mg of freeze-dried biomass were used for DNA extraction from reference strains. Genomic DNA from biomass of the reference strains and filtrides was extracted with the DNeasy Plant Mini Kit (QIAGEN, Hilden, Germany) according to the manufacturer’s instructions. For DNA extraction from filtrides, the method was slightly modified by adding twice the amount of Buffer AP1, RNase A and Buffer P3 to fully suspend the samples. During the initial steps of extraction, samples were homogenized using zirconia/silica disruption beads (0.5 mm) and by vortexing for 1 min. The extract was assessed spectrophotometrically (NanoDrop ND-1000, Thermo Scientific, Waltham, MA, USA), and the A260/A280 ratio varied between 1.22 and 2.04.

Qualitative PCR reactions were performed in order to detect the presence of the following cyanotoxin-coding genes: microcystin (MC; *mcyE*), cylindrospermopsin (CYN; *cyrJ*), saxitoxin (STX; *sxtG*, *sxtS*) and anatoxin (ATX; *anaC*) synthetase genes. A 20 µL PCR reaction mix containing 1× Phire Reaction Buffer, 0.4 µL Phire II HotStart polymerase (Thermo Scientific), 0.2 mM dNTPs (Thermo Scientific), 0.5 µL forward and reverse primers (Table 2), 2 µL of template and sterile deionized water was used. PCRs were run on a C1000 Touch Thermal Cycler (Bio-Rad) according to the following protocols: initial denaturation for 30 s at 98 °C; 40 cycles of 5 s at 98 °C, 5 s at 61 °C (for primers HEPF, HEPR), or 62 °C (for primers cyrJ_F, cyrJ_R, sxtG432_F, sxtG928_R, sxtS205_F, sxtS566_R), or 52 °C (for primers anaC-genF, anaC-genR) and 10 s at 72 °C; and a final extension of 1 min at 72 °C [16]. The following strains were used as a reference in the control template: PCC7820 for *mcyE*, CS-506 for *cyrJ*, CS-537/13 for *sxtG*, *sxtS* and *Dolichospermum* 123 for *anaC*. Visualization of PCR products was performed on a 1.5% Top Vision agarose gel (Thermo Scientific) dyed with SYBR^®^ Safe DNA gel stain. The observed bands were documented on Gel Doc™ XR (Bio-Rad, Helsinki, Finland) using Quantity One software (v. 4.6.9; 2004; Bio-Rad, Helsinki, FInland).

The reference strains mentioned above are the following: PCC7820 (*Microcystis aeruginosa*) as an MC producer, CS-506 (*Cylindrospermopsis* curr. *Raphidiopsis*) as a CYN producer, CS-537/13 (*Dolichospermum*) as a STX producer and SYKE-123 (*Dolichospermum*) as an ATX-a producer. These strains were obtained from the Pasteur Culture Collection (PCC), Australian National Algae Culture Collection (CS) and Finnish Environment Institute (SYKE).

### 2.6. Cyanotoxin Analyses

#### 2.6.1. Preparation of Water Samples for Liquid Chromatography–Tandem Mass Spectrometry (LC–MS/MS)

Preparation of water samples for LC–MS/MS was similar to the procedure described in Tokodi et al. [16]. In short, toxin concentration was determined by first filtering 100–250 mL of water samples depending on the bloom intensity through 2–3 µm filters. The biomass on the filter was freeze-dried and the filtrides placed in glass tubes. Toxin extraction was accomplished by firstly adding 3 mL of 75% MeOH and running the samples through 15 min of bath ultrasonication followed by 1 min of extraction with a Bandelin Sonopuls HD 2070 (Berlin, Germany) micro-tip probe sonicator (30% pulse and 30% energy). Extracts were then centrifuged at 10,000× *g* for 10 min at RT and the supernatants were evaporated to dryness in glass tubes (50 °C, nitrogen flow). Samples were then resuspended in 200 µL of 75% MeOH for microcystin (MC) analyses and in 200 µL of Milli-Q ultrapure water (Millipore, Molsheim, France) for cylindrospermopsin (CYN) analyses by vortexing and subsequently filtering (0.2 µm; GHP Acrodisc 13; Pall Corporation, Port Washington, NY, USA) into inserts. Samples were thus ready for LC–MS/MS analysis.

Filtrated water was concentrated by solid-phase extraction (SPE) on Water Oasis HLB (Wexford, Ireland). Samples were then eluted with 5 mL of 90% MeOH and 2 mL were transferred into glass tubes and evaporated using nitrogen flow. The pellet was then redissolved in 200 µL of 75% MeOH by vortexing, and subsequently filtered (0.2 µm; GHP Acrodisc 13; Pall Corporation, Port Washington, NY, USA) into inserts. Samples were thus ready for LC–MS/MS analysis.

#### 2.6.2. Preparation of Fish Tissue Samples for Liquid Chromatography–Tandem Mass Spectrometry (LC–MS/MS)

Depending on the size of the tissue, samples were either processed separately, or as whole entrails. Samples of the same organ of all the individuals of the same species were pooled together. The extraction procedure was similar to the one described by Tokodi et al. [16,27]. In short, samples were homogenized and freeze-dried. After freeze-drying, approximately 400 mg of homogenates were placed into a glass tube filled with 10 mL of 75% MeOH for cyanotoxin extraction overnight. Homogenization was then conducted for 30 s on ice, and the samples were ultrasonicated in a bath sonicator for 15 min, and additionally extracted with a probe sonicator (Bandelin Sonopuls HD 2070 micro-tip). Samples were then centrifuged for 10 min at 10,000× *g*. Ten milliliters of supernatant were then removed, and 5 mL of hexane were added. The lipid (hexane) layer was removed, and samples were concentrated by SPE (Waters Oasis HLB 30 mg) and eluted with 5 mL 90% MeOH. Two milliliters of each sample were then evaporated (50 °C, nitrogen flow), redissolved in 200 µL 25% MeOH and filtered (0.2 µm GHP Acrodisc 13; Pall Corporation, Port Washington, NY, USA) into inserts, thus ready for LC–MS/MS analysis.

#### 2.6.3. High-Performance Liquid Chromatography with Diode-Array UV Detection (HPLC–DAD)

HPLC–DAD was used for MC-related work. The reference samples, i.e., the samples used for identification and quantification purposes, as well as the unknown samples, were chromatographed on an Agilent (Waldbronn, Germany) 1100 series HPLC system composed of a vacuum degasser, a quaternary pump, a thermostated column compartment set at 40 °C and a diode-array detector operated at 200–300 nm (quantification wavelength 238 nm). The stationary phase was a Merck (Darmstadt, Germany) Purospher STAR RP-18e column, 55 mm × 4 mm I.D., with 3 µm particles. The mobile phase consisted of (solvent A) Milli-Q ultrapure water (Millipore, Molsheim, France) containing 0.05% trifluoroacetic acid (TFA; Fluka, Buchs, Switzerland) and (solvent B) acetonitrile (Fisher Scientific, Loughborough, UK) containing 0.05% TFA. The following linear gradient program was used: 0 min 25% B, 5 min 70% B, 6 min 70% B, 6.1 min 25% B; stop time 9 min; flow-rate 1 mL/min. The injection volumes were 10 µL.

MCs were identified with HPLC–DAD based on retention time and UV spectrum as detailed in the standard operating procedure SOP_TOXIC_AAU_06F [28]. These identifications were verified by LC–MS/MS data (Section 2.6.4 and Figure S1). The following reference samples were used: (a) extracts of *Microcystis* PCC 7820 (Pasteur Culture Collection, Paris) containing mainly MC-LR, MC-LY, MC-LW and MC-LF and (b) extracts of *Microcystis* NIES-107 (National Institute for Environmental Studies, Tsukuba, Japan) containing mainly MC-RR, MC-YR, MC-LR and their demethylated variants. The identities of MCs in the reference samples had been earlier verified by comparing chromatographic and spectral data to those of purified MCs (Meriluoto et al. [29]; Spoof et al. [30]; SOP_TOXIC_AAU_06F and SOP_TOXIC_AAU_09F in Meriluoto and Codd [28]). The calibration curves for MCs were constructed according to SOP_TOXIC_AAU_03F and SOP_TOXIC_AAU_06F [28]. HPLC–DAD chromatograms and UV spectra of MC-containing samples are presented in Figure S1.

#### 2.6.4. Liquid Chromatography–Tandem Mass Spectrometry (LC–MS/MS)

The theoretical and practical framework of LC–MS/MS experiments and examples of mass spectra in the context of cyanobacterial toxins have been recently presented by Caixach et al. [31]. The LC–MS/MS experiments of this paper were carried out on an Agilent 1200 Rapid Resolution (RR) LC coupled to a Bruker Daltonics HCT Ultra iontrap mass spectrometer (Bremen, Germany) with an electrospray ion (ESI) source as reported earlier [16,27,32]. The 1200 RR LC system consisted of a binary pump, a vacuum degasser, an SL autosampler and a thermostatted column compartment.

The separation of the MC samples was achieved on an Ascentis C18, 50 mm × 3 mm I.D. column with 3 µm particles (Supelco, Bellefonte, PA, USA) at 40 °C. The injection volumes were 5 µL. The mobile phase consisted of water–acetonitrile–formic acid (99 + 1 + 0.1; solvent A) and acetonitrile–formic acid (100 + 0.1; solvent B) with the following linear gradient program: 0 min 25 % B, 5 min 70 % B, 6 min 70 % B, 6.1 min 25 % B; stop time 10 min; flow rate 0.5 mL/min. Water was Milli-Q water purified to 18.2 MΩ.cm. Acetonitrile and formic acid were supplied by Fisher Scientific and Fluka, respectively. The following ion source conditions were used for the MS of MCs: positive electrospray ion mode, dry temperature 350 °C, nebulizer pressure 40 psi, dry gas flow 10.0 L/min, capillary voltage 4.0 kV. An MS scan range from *m*/*z* 500 to *m*/*z* 1200 with the Smart Parameter Setting (SPS) function was employed. The ICC target was set to 300,000 with a maximum accumulation time of 100 ms. MS/MS spectra were collected from *m*/*z* 180 to *m*/*z* 1200. Abundant MS–MS fragmentation was assisted by the Smart Frag setting. The quantification of MCs was based on the MS data of the protonated MC ions (doubly charged for MC-RR and its demethylated form, singly charged for other MCs). Calibration curves were prepared with diluted reference samples in which MCs had been quantified by HPLC–DAD (Section 2.6.3.). The identification was based on both MS data and MS/MS spectra. Data acquisition was done with Compass 1.3 software (Bruker Daltonics).

For CYN, the following changes in the LC–MS/MS conditions were applied: LC linear gradient program 0 min 0% B, 2.5 min 0% B, 2.6 min 50% B, 4 min 50% B, 4.1 min 0% B; stop time 10 min [33]. The MS scan range was from *m*/*z* 395 to *m*/*z* 440. The ICC target was set to 200000. An MS/MS fragmentation of the target mass *m*/*z* 416 was performed and the resulting MS/MS spectra were collected in the range *m*/*z* 150-440. The reference material for CYN was CRM-CYN from the Institute for Marine Biosciences (NRC-IMB, Halifax, Canada), used in dilutions.

LC–MS traces and MS/MS spectra related to MCs and CYN are presented in Figure S1.

### 2.7. Histological Analyses

#### 2.7.1. Sample Preparation

As mentioned above, tissue samples for histological analyses were collected and fixed in 10% neutral buffered formalin. Samples were processed by a standard histological procedure and as described in our previous studies [16,27,34]. In short, samples were dehydrated in graded series of EtOH, cleared in xylol and embedded into paraffin blocks. Prior to processing, gill and muscle samples were decalcified in 75% RDO Rapid Decalcifier solution (Apex Engineering Products Corporation). Three five-micron thin sections were cut per sample and stained with a standard hematoxylin and eosin (H&E) staining procedure. Sections were examined under a Nikon Eclipse 600 microscope and photographed using a QImaging Micro Publisher 3.0 digital camera.

#### 2.7.2. Semi-Quantitative Analysis

In order to ensure comparations of histopathological lesions among different seasons and fish species, we have utilized a semi-quantitative scoring system proposed by Bernet et al. [35] for quantifying lesions of hepatopancreas, kidneys and gills. The protocol was slightly modified by omitting inflammation and tumors, and focusing solely on progressive, regressive and circulatory disturbances (Table S1). The relevance and pathological importance of each alteration (i.e., how the organ function is affected by the alteration) was expressed by an importance factor ranging from 1 (minimal importance; lesion is reversible after the irritant ends) to 3 (marked importance; lesion is generally irreversible) (Table S1). The prevalence of each alteration was assessed by a score value ranging from 0 (unchanged) to 6 (severe occurrence). Alteration indices were calculated by multiplying the importance factor and score value for the given alteration. The organ index was calculated by summarizing all alteration indices for the given organ, while the total index for a given fish was calculated by summarizing all organ indices for the given fish. Organ indices were compared between sites by using one-way ANOVA, followed by a Tukey’s HSD post hoc test.

## 3. Results

### 3.1. Physical and Chemical Parameters of Water

In Lake Balaton, all of the investigated water quality parameters were within the guidelines proposed by the Hungarian Government Decree [36] (Table 3). In the KBWPS, several values were outside of the proposed guidelines, and all were indicative of a mass blooming event. pH levels were alkaline during the whole investigated period. Oxygen levels (depicted in total dissolved oxygen and oxygen saturation) were normal in April, however, they started to drop significantly until reaching the lowest point in September. High ammonium levels in September, as well as high orthophosphate levels in April and September, are indicative of high organic load within the KBWPS, as well as of a blooming event occurring in the reservoir.

### 3.2. Qualitative and Quantitative Analyses of the Cyanobacterial Community

In the KBWPS, *Aphanizomenon flos-aquae* Ralfs ex Bornet and Flahault was the most dominant species observed in all investigated seasons (Table 4). Additionally, *Dolichospermum spiroides* (Klebhan) Wacklin, L. Hoffmann and Komárek and *Microcystis aeruginosa* (Kützing) Kützing were quite numerous. When looking at the total number of cells/mL, in all seasons, the number of cells exceeds the guideline of 10,000 proposed by Falconer [37], indicating a cyanobacterial bloom. The highest number of cyanobacteria was detected during July, while the lowest numbers were detected in April.

In Lake Balaton, only two cyanobacterial species, *Microcystis aeruginosa* (Kützing) Kützing and *Oscillatoria tenuis* C. Agardh ex Gomont, were detected. Even though their numbers were bloom-worthy, they were still far lower than the numbers observed in the KBWPS. The invasive species *Cylindrospermopsis raciborskii* (curr. *Raphidiopsis raciborskii*) was not detected in neither KBWPS nor Lake Balaton.

### 3.3. Detection of Cyanotoxin-Coding Genes

**Figure 2:**
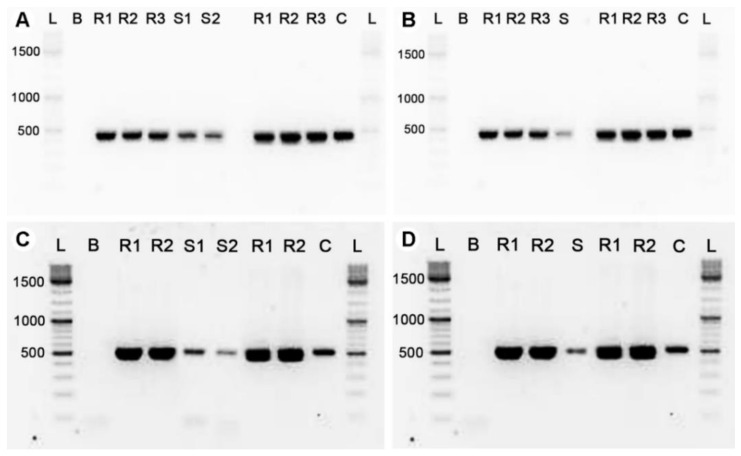
Visualization of the PCR products for microcystin synthetase (*mcyE*) from KBWPS (**A**) and Lake Balaton (**B**), as well as for saxitoxin synthetase (*sxtG*) from KBWPS (**C**) and Lake Balaton (**D**). L: ladder; B: blank; S: sample from June 2018 (Lake Balaton); S1: sample from July 2018 (KBWPS); S2: sample from September 2018 (KBWPS); R1-R3: reference strains; C: exogenous amplification control.

In both July and September 2018 in the KBWPS, and in June 2018 in Lake Balaton, we detected the presence of MC- (*mcyE*; Figure 2A,B) and STX-coding (*sxtG*; Figure 2C,D) genes in the filtered biomass. The presence of the *stxS* gene as well as CYN- (*cyrJ*) and ATX-coding (*anaC*) genes was not detected in this study.

### 3.4. Presence of Cyanotoxins in Water and Fish Tissues

Cyanotoxin content in water was tested for the presence of cyanotoxins in the KBWPS during July and September 2018, and Balaton during June 2018. In the KBWPS, five MC variants were detected, out of which MC-LR had the highest concentration (Table 5). CYN was not detected. In Lake Balaton, tested cyanotoxins were not detected in water. Additionally, tested cyanotoxins were not detected in sampled fish tissues in any of the sites, nor seasons.

### 3.5. Histopathological Alterations of Fish Tissues

During the whole investigated period, different organs of fish caught at the KBWPS and Lake Balaton were checked for the presence of tissue damage through histopathological analyses. During different seasons, different fish species were caught (Table 1). However, as all fish belonged to the same family (Cyprinidae), they all occupy a similar space within the aquatic food chain, and most importantly, as we did not detect any differences in histopathological indices between different species within a season (when possible), the results are presented as combined for all species. No significant alterations were observed in spleen, gonads and muscle.

#### 3.5.1. Hepatopancreas (Liver)

**Figure 3:**
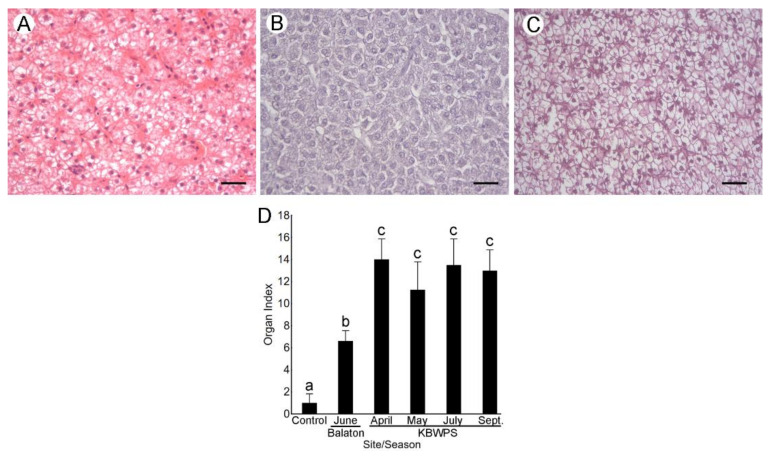
Histopathological alterations of the hepatic tissue of fish caught from Lake Balaton and Kis-Balaton Water Protection System (KBWPS). (**A**) Control individuals demonstrating normal hepatic histology. (**B**) Individuals caught from Lake Balaton displaying severe basophilia and rounding of cells. (**C**) Individuals caught from KBWPS showing pronounced vacuolization, loss of glycogen as well as pyknosis. H&E staining. Scale bars: A and B: 25 µm, C: 50 µm. (**D**) Hepatopancreas indices obtained after the semi-quantitative analysis of the histopathological alterations. Different letters above the SD bars indicate statistical significance (Tukey’s HSD, *p* < 0.05).

Control individuals displayed a normal cord-like parenchymal structure as hepatocytes were organized in cords around blood vessels and sinusoids (Figure 3A). Hepatocytes had a normal polygonal shape, and nuclei were visible with prominent nucleoli. Very slight vacuolization of hepatocytes was noticed in some of the controls.

The predominant alteration observed in the hepatic tissue of fish from Lake Balaton was a severe basophilia of hepatocyte cytoplasm (Figure 3B). Slight loss of the cord-like parenchymal structure and rounding of cells were also noted. On the other hand, fish from the KBWPS displayed severe vacuolization and loss of glycogen as cells appeared transparent (Figure 3C). This consequently led to hypertrophy, i.e., an increase in cell size. Loss of the cord-like parenchymal structure and rounding of cells could be observed, however, it was not as prominent. Nuclear alterations were observed as well; nuclei of many cells appeared pyknotic as the chromatin was very condensed, and nucleoli could be hardly observed. All mentioned histopathological changes were observed throughout the seasons with a similar intensity. The only difference was a slightly higher occurrence of architectural changes (primarily loss of the parenchymal structure) in fish caught during September.

For the semi-quantitative analysis, loss of the cord-like parenchymal structure and rounding of cells were considered as architectural and structural alterations, while basophilia, vacuolization and glycogen loss were considered as plasma alterations of the hepatic tissue. This analysis corroborated qualitative observations. Organ indices depicting tissue alterations were the highest in fish from the KBWPS irrespective of the season (Tukey’s HSD, *p* < 0.01; Figure 3D). Even though organ indices of fish Lake Balaton were significantly lower than those from fish caught in the KBWPS, they were still higher than the controls (Tukey’s HSD, *p* < 0.05).

#### 3.5.2. Kidney

**Figure 4:**
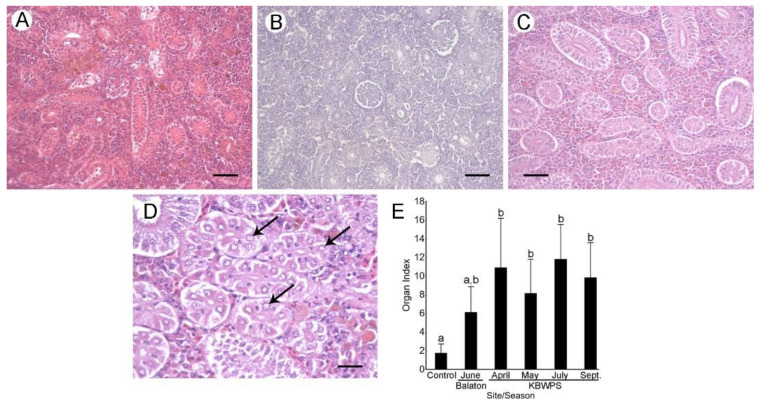
Histopathological alterations of the kidneys of fish caught from Lake Balaton and Kis-Balaton Water Protection System (KBWPS). (**A**) Control individuals demonstrating normal kidney histology. (**B**) Individuals from Lake Balaton showing slight tubular vacuolizations. Individuals from KBWPS displayed severe tubular vacuolizations leading to detachment from the basal membrane (**C**) as well as nuclear alterations, primarily karyolysis (**D**; arrows). H&E staining. Scale bars: (**A**–**C**): 50 µm, **D**: 25 µm. (**E**) Kidney indices obtained after the semi-quantitative analysis of the histopathological alterations. Different letters above the SD bars indicate statistical significance (Tukey’s HSD, *p* < 0.05).

Kidneys of control individuals displayed normal renal histology (Figure 4A). Proximal renal tubules had one layer of tall columnar epithelial cells with basal nuclei, while distal tubules contained one layer of shorter epithelial cells with central nuclei. Renal corpuscles had a relatively large glomeruli with a thin capsular space of the Bowman’s capsule. Slight vacuolization of tubular epithelial cells was observed in some of the controls.

Kidneys of fish caught in Lake Balaton displayed primarily tubular alterations (Figure 4B). Some of tubules were vacuolated, and displayed nuclear alterations in the form of karyolysis where the chromatin was almost dissolved. These alterations were far more prominent in fish from the KBWPS as most of the tubular cells were severely vacuolized, even leading to detachment from the basal membrane (Figure 4C). Nuclear alterations were also prominent (Figure 4D). Additionally, alterations of the glomeruli could be observed as the mesenchymal cells and the visceral layer of the Bowman’s capsule looked smaller and atrophied, which in turn led to dilatation of the capsular space. All mentioned alterations were noted in all investigated seasons, and there were no differences in frequencies of any of the alterations between seasons.

For the semi-quantitative analysis, vacuolization of tubular cells and their detachment from the basal membrane were considered architectural and structural alterations of the tubules, while dilatations of the Bowman’s capsule were considered as architectural and structural alterations of the glomerulus. Organ indices of fish from the KBWPS were significantly higher than those from the control individuals (Tukey’s HSD, *p* < 0.05). Fish from the KBWPS also displayed higher indices than fish from Lake Balaton, however, differences were not statistically significant. The highest average kidney organ index was observed in July which corresponded to the highest cyanobacterial concentrations and highest observed MC concentration; however, significant differences between the seasons were not observed.

#### 3.5.3. Gills

**Figure 5:**
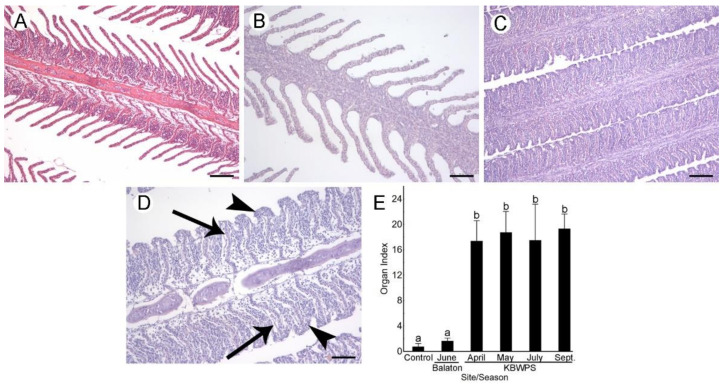
Histopathological alterations of gills of fish caught from Lake Balaton and Kis-Balaton Water Protection System (KBWPS). (**A**) Control individuals demonstrating normal gill histology. (**B**) Individuals from Lake Balaton showing slight tubular clubbing of lamellae and occasional aneurysms. (**C**) Individuals from KBWPS displayed severe hyperplasia of the interlamellar cell mass which led to a complete fusion of lamellae. Occasionally, fusions were also coupled with epithelial lifting (**D**; arrows) and epithelial hypertrophy (**D**; arrowheads). H&E staining. Scale bars: (**A**,**C**,**D**): 100 µm, **B**: 50 µm. (**E**) Gill indices obtained after the semi-quantitative analysis of the histopathological alterations. Different letters above the SD bars indicate statistical significance (Tukey’s HSD, *p* < 0.05).

Gills of control individuals displayed normal gill histology (Figure 5A). Large primary lamellae were regularly lined with secondary lamellae on both sides. Secondary lamellae had one layer of epithelial cells, and their diameter could enable circulation of single-erythrocyte streams. The interlamellar cell mass contained epithelial cells and fewer supportive (chloride and mucous) cells. Slight epithelial lifting (predominantly at the base of secondary lamellae) was observed in some of the controls.

Gills of fish from Lake Balaton displayed very mild alterations. These alterations consisted of clubbing of lamellae, slight epithelial hypertrophy and infrequent hyperemia (Figure 5B). Gills of fish from the KBWPS on the other hand showed more prominent alterations. All fish, irrespective of the season, exhibited hyperplasia of the interlamellar cell mass which frequently led to complete fusions of secondary lamellae (Figure 5C). Furthermore, this proliferation coincided with lifting of the epithelium and hypertrophy of epithelial cells (Figure 5D). Hyperemia characterized by an increased blood flow through the secondary lamellae was also prevalent. All mentioned alterations were noted in all investigated seasons.

For the semi-quantitative analysis, epithelial lifting, clubbing of lamellae and rupture of epithelium were considered architectural and structural alterations of the epithelium. Gill indices of fish from the KBWPS were significantly higher than indices of fish from Lake Balaton and control fish (Tukey’s HSD, *p* < 0.05). No differences were observed between seasons.

## 4. Discussion

Lake Balaton has been a focus of numerous investigations which demonstrated a deterioration and subsequent amelioration of its water quality after several remedial steps were undertaken. Most of these studies have been focused on the water quality and cyanobacterial community of Lake Balaton [1,6,15,38,39,40]. Here, for the first time, we have detected the presence of five out of nine tested microcystin congeners in the water of the KBWPS, while no toxins were detected in Lake Balaton. We have also observed the presence of MC- and STX-coding genes in the cyanobacterial biomass of both ecosystems. Lastly, histopathological analyses displayed severe hepatic, kidney and gill alterations in fish obtained from the KBWPS, while fish from Lake Balaton displayed milder alterations and more resembled the structure of control fish. Below, the observed water quality parameters, cyanobacterial bloom occurrence and its effects on fish tissues will be discussed. Lastly, we will review the ability of the KBWPS to prevent toxic cyanobacterial blooms from spilling into Lake Balaton.

### 4.1. Water Quality and Cyanobacterial Blooming

During June 2018, most of the water parameters observed in Lake Balaton were within proposed guideline limits. These results coincide with the results of Sebestyén et al. [41]. Values of pH, conductivity, dissolved oxygen and oxygen saturation are near the guideline values specified by the Hungarian Government Decree [36]. Levels of nitrates, nitrites and orthophosphates for Lake Balaton are difficult to discuss as the guideline levels are below our detection levels. However, the study of Sebestyén et al. [41] does indicate that all of these nutrients are in higher concentrations than the proposed guidelines, and that Lake Balaton is primarily contaminated with higher nitrate, Ortho-P and total phosphate levels. Interestingly, the highest concentrations of these nutrients were observed in Basins 2 and 3, and not in Basin 1 as would be expected, indicating that the Zala River is not the main contributor to these high values as in previous decades [17,41], and that they could be the result of diffuse pollution from the surrounding agricultural areas and wastewater, or of the internal load of the lake.

In the case of the KBWPS, several parameters were not within the proposed guidelines, and all were indicative of a mass blooming event. Indeed, the reservoir was in a constant bloom according to the number of cyanobacteria observed. Even the lowest number of cyanobacteria observed in April exceeded 10,000 cells/mL 30-fold, indicating a cyanobacterial bloom [37]. In May, this number increased an additional 20-fold, and it peaked in July after doubling in number since May. However, in September, the bloom started to collapse as the number of cyanobacteria decreased by three quarters. During the whole investigated period, pH was above the proposed guideline of 9. High water alkalinity has many times been associated with cyanobacterial blooms [42,43,44]. As for dissolved oxygen and oxygen saturation, these values were within the guidelines during April, however, they started to drop significantly in May, while reaching their lowest values in September. During the height of a bloom, pH and oxygen levels are usually high as a consequence of high photosynthetic activity. However, when the bloom breaks down, both of these levels drop, especially dissolved oxygen, as bacteria that digest the dead cyanobacterial and algal cells need oxygen for decomposition. Oxygen concentrations were low during the whole investigated period, while the lowest values were observed in September, which concurred with the decrease in the number of cyanobacterial cells (i.e., bloom decay).

Nitrogen needed for protein synthesis and phosphorus needed for DNA/RNA synthesis and energy transfer are the two most important nutrients required for an intense cyanobacterial growth and are considered to be key limiting nutrients in many ecosystems [45,46,47,48,49]. As nitrate and nitrite concentrations of Lake Balaton were higher than the proposed guidelines [8], we presume that the concentrations of the KBWPS were also higher than the guidelines, even though these levels were still below our detection limits. Orthophosphates (PO_4_^3−^; Ortho-P) are usually the predominant inorganic P form and are, in particular, a determining factor in eutrophication as they are the form that is the most bioavailable for cyanobacteria to promote their primary growth [50,51]. Ortho-P was very high in April and September, while the levels were barely detectable during the two months in between. The concentrations observed in April coincided with the lower numbers of cyanobacteria, and most likely originated from the organic load of the Zala River. We postulate that the low concentrations of Ortho-P in the following month are due to a 20-fold increase in the number of cyanobacteria, where Ortho-P was probably the one of the main nutrients driving such proliferation. As the concentrations remained low in July (two months later), but cyanobacterial numbers doubled, cyanobacteria most likely used other P forms for proliferation. A great increase in Ortho-P levels occurs again in September as the number of cyanobacteria decreases and the bloom starts to decay. During algal decomposition, phosphate is again released from the cells, predominantly in the form of Ortho-P (sometimes even up to 96%) [52].

### 4.2. Cyanobacterial Community Structure

During June 2018, *Microcystis aeruginosa* and *Oscillatoria tenuis* were the only two cyanobacterial species detected in the Siófok Basin (Basin 4). This result differs from the observations of Tóth et al. [53] made during August 2018 and Farkas et al. [40] made during June 2017. In the study of Farkas et al. [40], the dominant species in the central and eastern basins (Basins 3 and 4) of Lake Balaton was determined to be the picocyanobacterium *Synechococcus* sp. The study of Tóth et al. [53] further confirms this finding as samples originating from Tihány were dominated by species originating from the order Synechococcales, while there were far fewer cyanobacteria from the order Nostocales. In addition, the study of Somogyi et al. [54] reviews the decade-long occurrence of picoplankton in Lake Balaton. The difference in cyanobacteria community composition between the studies most likely originates from the method of determination. In the present study, we used a traditional microscopy-based species determination method, whereas the studies of Tóth et al. [53] and Farkas et al. [40] solely relied on next-generation sequencing. Picocyanobacteria are very small (up to 2 µm in diameter [54]) and hardly detectable with light microscopy. As the present study focuses on toxic cyanobacteria, and given that the toxin-producing capabilities of picocyanobacteria are largely unknown, we have focused solely on microcyanobacteria which are known to have significant cyanotoxin production capabilities, and are generally considered the main species in toxin production. However, this discrepancy does highlight the utility of sequencing techniques in species determination, and can be used as a valuable complement to traditional methods.

In the KBWPS, intense cyanobacterial blooms observed throughout the investigated period were dominated by *Aphanizomenon flos-aquae*, with its contribution reaching up to 78% of the total cell numbers observed in July 2018. Other species, such as *Aphanizomenon hungaricum*, *Dolichospermum flos-aquae* (ex *Anabaena flos-aquae*), *Microcystis aeruginosa* and *Microcystis flos-aquae*, contributed to a far lesser extent. A similar situation was observed in the blooms of 2009 when *Aphanizomenon flos-aquae* dominated the bloom, with its biomass exceeding 70% in the sample, followed by *Cylindrospermopsis raciborskii* (curr. *Raphidiopsis raciborskii*) and *Anabaena* sp. [55]. As the KBWPS does not retain 100% of the phytoplankton and a part of the phytoplankton spills over into Lake Balaton, detection of some of these species, especially *Aphanizomenon flos-aquae*, in Basin 1 (Keszthely Basin) was expected. Indeed, the study of Farkas et al. [40] reports an abundance of *Aphanizomenon flos-aquae* in Basin 2 (Szigliget Basin), while the study of Tóth et al. [53] demonstrate a predominance of species belonging to order Nostocales in Basin 1. Furthermore, Farkas et al. [40] report the presence of *Aphanizomenon*, *Anabaena* and *Microcystis* genera in the Zala River which completely corresponds with the results of the present study.

An important finding of the study is the lack of *Cylindrospermopsis raciborskii* (curr. *Raphidiopsis raciborskii*) in both the KBWPS and Lake Balaton. This invasive species was first introduced into Lake Balaton in 1978 and has dominated the cyanobacterial community, causing many blooming events during the 1990s [56,57]. Mass proliferation of this species is concerning as it can release a potent cyanotoxin named cylindrospermopsin (CYN), even though CYN was not detected in strains growing in Europe [58,59]. Strains isolated from Lake Balaton did not produce cylindrospermopsin, nor anatoxin; however, aqueous extracts of the strain did exert toxicity according to a *Thamnocephalus platyurus* acute lethality test, *Daphnia magna* acute immobilization assay, *D. magna* feeding inhibition assay and *Danio rerio* embryo developmental toxicity assay [60].

### 4.3. Occurrence of Cyanotoxin-Coding Genes and Cyanotoxins

Apart from causing alterations in water quality and affecting nutrient loads of a given ecosystem, the main danger of cyanobacterial blooms comes from their ability to produce toxic secondary metabolites known as cyanotoxins. Cyanotoxins are very diverse in their structure and mechanism of toxicity [13,14]. However, a commonality amongst them all is a pronounced toxicity, and when released as a consequence of a cyanobacterial bloom, they could represent an imminent health hazard to other microorganisms, plants, animals, and also humans. Toxic effects and potential hazards have been reviewed elsewhere [8,61,62].

Among cyanotoxins, MCs are the most commonly detected and most widespread in freshwater ecosystems [61]. They are cyclic heptapeptides with a large array of congeners (more than 279 have been identified to date [63]). The occurrence of nine congeners has been surveyed in the water of the KBWPS and Lake Balaton for the first time; amongst them, MC-LR and MC-RR are considered to be the most studied and most commonly detected. Our analyses demonstrate no presence of any of the tested MC congeners in the water of Lake Balaton. On the other hand, a low presence of MCs was detected in the water of the KBWPS during July and September. Additionally, a significantly higher concentration of 1.29 µg/L was detected in July 2018 which coincided with the peak of the bloom where the total number of cyanobacterial cells per milliliter reached almost 14 million. Coincidentally, MC-LR concentration detected in September was decreased to a quarter compared to the values detected in July, similarly to the number of cells, which had also decreased to a quarter during that period. It was not possible to identify which species were responsible for the MC-LR production, as all species dominating the bloom (including *Aphanizomenon*, *Dolichospermum* and *Microcystis*) were previously known to have the ability to produce MCs. However, after observation of the numbers of each species individually, only cell numbers of *Microcystis flos-aquae* decreased to a quarter from July to September, while cell numbers of other species decreased at a higher or lower rate than a quarter. Furthermore, the presence of MC-coding genes (*mcyE*) and saxitoxin-coding genes (*stxG*) was detected in both the KBWPS and Lake Balaton, with *Microcystis flos-aquae* being the only species detected at both sites. However, *Microcystis* is not known to produce saxitoxin; this was only detected in the genera *Aphanizomenon*, *Anabaena*, *Planktothrix*, *Cylindrospermopsis* (curr. *Raphidiopsis*), *Lyngbya* and *Scytonema* [64]. In addition, species dominating the bloom in the present study are known for producing other toxins such as anatoxin-a, β-N-methylamino-L-alanine (BMAA), aeruginosin, cyanopeptolin, microcyclamide, microguanidine, microviridin and various lipopolysaccharides [10,14,65,66], therefore, monitoring of the investigated sites for other toxins and congeners is of paramount importance. The detection of MC and STX synthetase genes within the biomass of cyanobacteria isolated from both sites further demonstrated the need for regular monitoring as this finding presents a latent threat that potential future cyanobacterial blooms can become toxic.

### 4.4. Effects of Cyanobacterial Blooming on Fish Tissues

Fish are at the top of the food chain in aquatic ecosystems and have an important role in maintaining the stability of these ecosystems. As such, fish are commonly exposed to cyanobacteria, especially planktivorous or phytoplanktivorous fish, as cyanobacteria are an important component of their diet. Apart from providing nutritive value, cyanobacteria may exert negative effects on fish during blooming events as they can modify the environment and water quality, as well as produce toxic secondary metabolites [67]. Damage in fish tissues can occur from high water alkalinity, hypoxia of high ammonia concentrations or from the direct action of cyanotoxins.

The hepatopancreas is a primary target organ for many cyanotoxins, especially the most commonly produced hepatotoxin, MC. MCs usually enter the bloodstream after ingestion of cyanobacteria directly [68], while exposure of piscivorous fish through biomagnification is still under debate [69]. Once MCs enter the hepatocytes through the organic anion-transporting polypeptide superfamily (OATPs) [70], they start to exert their toxic effects mainly through covalent binding to serine–threonine protein phosphatases (PP1 and PP2A) [71,72]. Through this action, MCs can impair many cell functions such as cytoskeletal stabilization, DNA repair, immune response, initiation of apoptosis, genotoxicity and others [73].

The most common histopathological alterations observed after exposure of fish to cyanotoxins are loss of the parenchymal cell structure, rounding of cells, vacuolization, loss of glycogen and pyknosis [67]. All of these alterations were observed in the present study to varying degrees depending on the season and locality. The main alteration in the hepatic tissue of fish from Lake Balaton was intense basophilia which is primarily associated with an increased mRNA content, usually localized within the rough endoplasmic reticulum or Golgi apparatus, and is indicative of the synthesis of protein destined to be secreted [74]. On the other hand, the most pronounced alteration in the hepatic tissue of fish from the KBWPS was intense vacuolization characterized by diffuse microvesicular fatty accumulation within the cytoplasm, which is indicative of a more serious hepatic dysfunction, and is usually a reflection of toxicity and/or nutritional disturbances [75]. Vacuolization, loss of the cord-like parenchymal structure, rounding of cells and pyknosis were observed in cyprinid fish exposed to cyanobacterial blooms in natural conditions [16,27,34,76,77,78]. As the KBWPS was blooming throughout the investigated period, and a similarity between alterations detected in different fish species from the KBWPS and other studies is apparent, we can postulate that hepatic damage observed in fish from the KBWPS could be a consequence of cyanobacterial blooming.

As kidney tubule cells possess multi-specific organic anion-transporting polypeptides (OATPs), similar to hepatocytes [79], MCs can enter renal epithelial cells and through binding to PPs cause many alterations, as previously stated [80]. Indeed, fish exposed to MCs did display alterations in the renal tissue, primarily in the form of tubule vacuolization of the tubular epithelial cells, glomerular atrophy or necrosis, dilatation of the Bowman’s capsule and others [67]. Similar alterations were observed in the present study, as well as in other blooming ecosystems [16,27,34,77,81]. Renal alterations were stronger in the KBWPS than in Lake Balaton, however, statistical delineations could not be observed. The highest renal index was observed in July in the KBWPS which coincided with the largest number of cyanobacterial cells present, and the highest MC-LR concentration detected. As Fischer and Dietrich [82] have elucidated that MC-induced kidney pathologies in cyprinids can develop rapidly and at lower concentrations, the alterations observed in the present study could most likely be caused by the cyanobacterial bloom and cyanotoxins present within the blooming water.

The gills of fish are in direct contact with water, and are therefore considered a valuable marker for environmental pollution [35,83]. Gills of fish from Lake Balaton did not display any significant alterations and were similar to the control. On the other hand, during the investigated period, gills of different fish species from the KBWPS displayed severe alterations such as hyperplasia of the interlamellar cell mass, which frequently led to a complete fusion of secondary lamellae, epithelial hypertrophy, epithelial lifting and hyperemia. These alterations were frequently observed in gills of fish exposed to cyanobacterial blooming in natural conditions [16,27,34,67]. The predominant gill alterations of fish from the KBWPS, which were hyperplasia and epithelial lifting, are defensive mechanisms which reduce the uptake of xenobiotics by reducing the respiratory surface area, or increasing the water–blood barrier [83]. Additionally, in gills, we can see a different reaction pattern than in the hepatopancreas and kidneys. The hepatopancreas and kidneys mostly displayed regressive changes which led to a functional reduction of the organs. These changes (such as architectural alterations, atrophy, necrosis) are mostly associated with direct damage caused to an organ. On the other hand, gills of fish from the KBWPS displayed mostly progressive changes that lead to an increased activity of the organ. Hyperplasia, which presents as the proliferation of epithelial cells, is indicative of an increased activity of the gills, however, when it leads to complete fusions of the lamellae, it can drastically reduce the respiratory surface of the gills, and thus significantly hinder their function. Even though alterations observed in the gills are not indicative of direct toxin action, nor organ damage, they still represent a defensive action of the organ towards the xenobiotics present within the water, and can be caused by cyanobacterial blooming. Furthermore, a drastic reduction of the respiratory surface caused by fusion of secondary lamellae caused by the bloom can impair the functionality of this organ, and can lead to further damage in other organs and tissues caused by hypoxia.

Apart from the descriptive analyses of histopathological alterations, in the present study, we have employed a semi-quantitative analysis to partially quantify the described alterations. Through this analysis, we were able to gain more insight into the severity of the observed alterations and could compare them between seasons and ecosystems. This type of analysis is particularly useful in field studies when different species are sampled, and direct quantification of cell size or shape would not be useful due to species-specific or size-specific differences between individuals. It is important to point out that in the present study, most of the individuals were of similar size (Table 1) and all belonged to the Cyprinidae family. Additionally, all sampled species are omnivorous, occupy a similar space within the aquatic food chain and are affected by similar nutritional or ecological factors (e.g., cyanobacterial blooming), therefore, no differences in alterations between the species were noted.

Cyanotoxins are also known to accumulate within fish tissues [84]. The study of Flowes et al. [84] displayed that omnivorous fish species accumulate the most cyanotoxins, even more than planktivorous or phytoplanktivorous fish. As previously stated, all analyzed fish were omnivorous, however, no toxin accumulation was noted. It is possible that the toxin concentrations in the water were not high enough to cause accumulation in fish tissues, as a correlation between the two was observed [84]. On the other hand, there are many challenges in the analyses of cyanotoxins in animal and plant tissues or, e.g., soil. A detailed discussion of the topic is beyond the scope of the present paper but a brief summary of these challenges follows. The metabolism of MCs in the exposed organisms is not fully understood and there is likely at least some inter-species variation in this respect. It is recognized that MCs can be covalently bound to cysteine residues in some cellular proteins, making the MCs non-extractable in a procedure similar to that used in the current paper. There is also the possibility of conjugate forming between MCs and glutathione as a part of the organisms’ detoxification strategies [85,86]. Further, complex matrices such as tissues or soil tend to show non-optimal recoveries in the extraction processes due to interfering compounds or adsorption to the matrix constituents. Finally, ion suppression or enhancement due to co-eluting compounds in mass spectrometric detection may affect the quantitation. We studied the recovery of spiked MCs in the extraction process involving fish tissues and found it to be 34–94% [34]. A lower recovery was characteristic for more hydrophilic MCs (MC-RR and its demethylated form). Even though there are many challenges in the analyses of cyanotoxin accumulation in tissues, further monitoring is necessary as oral ingestion of cyanotoxins accumulated in fish is one of the potential exposure routes of humans to these toxins [87].

### 4.5. Safeguarding Properties of KBWPS

As Lake Balaton is an important tourist attraction and leisure/recreational center, safeguarding good water quality and prevention of eutrophication is of essential importance for this lake. Until the 19th century, this was possible as anthropogenic activity was very limited, and a wetland named the Kis-Balaton Wetland (KBW) formed at the delta of the Zala River into Lake Balaton filtered the majority of the organic load coming from the Zala River, thus ‘protecting’ Lake Balaton. However, during mid- and late 19th century, and especially during the mid-20th century, the water quality of Lake Balaton started to severely degrade due to anthropogenic activity, such as channelization, heightened agricultural activity and the use of phosphorus- and nitrogen-rich fertilizers, sewage effluents of growing coastal towns and others [1]. In order to mitigate the significant eutrophication that started to occur, management steps such as sewage diversion from major coastal cities, the creation of waste water treatment plants (WWTPs) in Zalaegerszeg and Keszthely which would remove P from the wastewaters, downsizing of large livestock farms and reconstruction of the KBW (now named the KBWPS) have been conducted [5,17].

**Figure 6:**
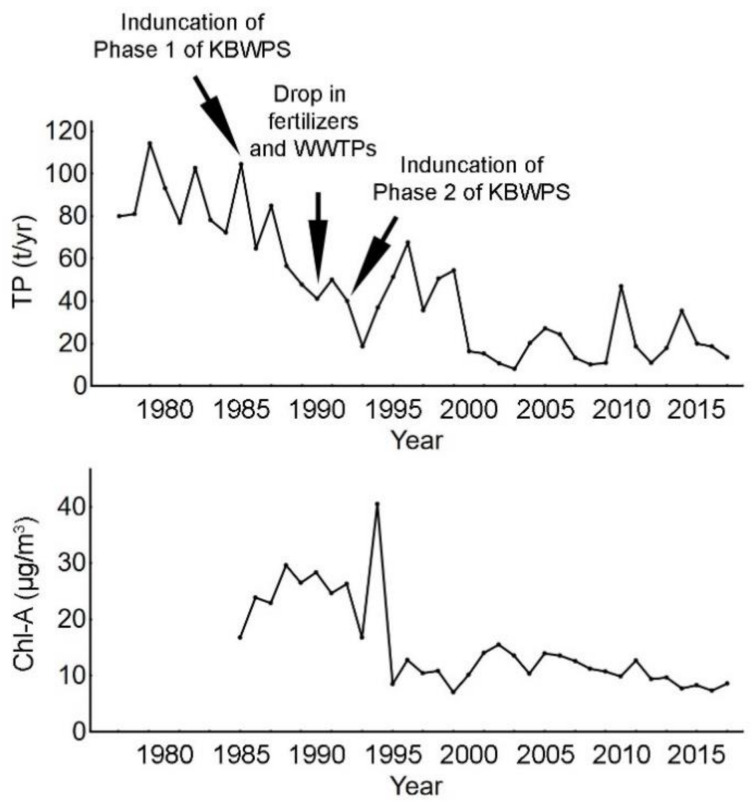
Average annual concentrations of total phosphorus (TP) and chlorophyll a (Chl-a) in Lake Balaton during the period from 1977 to 2017. Figure is based on that in Hatvani et al. [17] with permission from the publisher.

Studies on the temporal changes in the trophic state and nutrient load of Lake Balaton demonstrate several key moments and time periods that affected the lake. The period from 1985 to 1994–1995 is characterized by an intense and sharp decrease in average annual chlorophyll a (Chl-a) levels and a significant decrease in the total nitrogen levels, total phosphorus levels and soluble reactive phosphorus (Figure 6) [5,15,17]. The initial oligotrophication began after the inundation of Phase I of the KBWPS (Hídvégi Pond), and it continued significantly during the early 1990s with the drop in phosphate-based fertilizers, the introduction of WWTPs and the partial inundation of Phase II of the KBWPS (Fenéki Pond). Even though the drop in total nitrogen, total phosphorus and soluble reactive phosphorus levels was immediate following these management strategies, the decrease in Chl-a and oligotrophication occurred with a certain time lag. The significant drop in Chl-a levels occurred in 1995 and this pushed the trophic state of the lake from hypereutrophic to eutrophic [5,15,17]. Spatial differences in response were also evident [17]. Oligotrophication of Basin 1 (Keszthely basin), which is located at the mouth of the Zala River, was severe in response to the construction of the KBWPS as its trophic state was directly influenced by the inflow of the Zala River, while a more delayed response was observed in Basins 3 and 4 (Szemes and Siófok Basins) and was influenced by the reduction in fertilizer use and construction of WWTPs. When taking into account the historical data of average nutrient and Chl-a values, there is an apparent effect of the inundation of the KBWPS on the amelioration of Lake Balaton’s water quality.

Currently, due to climate change and hydro-meteorological conditions, the Zala River flows into Lake Balaton only during winter and spring, therefore, its effect on the water quality of the lake is becoming limited. At this timepoint, the internal load of the lake and diffuse pollution from sewage effluents and agricultural areas have a more pronounced effect on the trophic status of the lake. This is corroborated by the study of Sebestyen et al. [41] which demonstrates that the inner basins (predominantly Basins 2 and 3) have a higher total phosphorus, Ortho-P and nitrate concentrations than Basin 1. However, this does not take away from the importance of the KBWPS, as it considerably reduces the nutrient load flowing in from the Zala River.

As demonstrated in many previous studies, the construction of the KBWPS had a significant effect on the reduction of nutrient loads and oligotrophication of Lake Balaton. A similar result was found in the present study. Water quality parameters of the KBWPS were indicative of intense cyanobacterial blooms and some parameters were outside of the proposed guidelines, while these parameters were within the proposed guidelines in Lake Balaton. While the KBWPS displayed very high cyanobacterial concentrations in the peak of the blooming season (~13 million), the numbers were much lower in Lake Balaton (~30,000) during the same timeframe. Additionally, MCs were detected in the KBWPS, with a peak value of 1.29 µg/L observed in July, while MCs were not detected in Lake Balaton. Additionally, fish from the KBWPS demonstrated severe histopathological alterations in the hepatopancreas, kidneys and gills, many of which could be directly caused by cyanotoxin action, while alterations were much less severe in fish from Lake Balaton and more closely resembled the normal structure observed in the controls. Nonetheless, it is important to note that the presence of MC- and STXsynthetase-coding genes was observed in the filtered biomass of both ecosystems, therefore, if a mass cyanobacterial blooming does occur in Lake Balaton, it could be potentially toxic and hazardous for all users of this essential aquatic ecosystem.

## 5. Conclusions

In the present study, we demonstrated severe cyanobacterial blooming in the KBWPS, with cell numbers reaching almost 14 million cells/mL at the peak of the bloom in July 2018. Five out of nine tested MC congeners were detected at the peak of the bloom, with the concentrations of MC-LR reaching 1.29 µg/L; however, CYN was not detected. Furthermore, MC- and STX synthetase-coding genes were detected in the cyanobacterial biomass obtained from the KBWPS. Histopathological analyses displayed severe hepatic, kidney and gill damage in different fish species obtained from the KBWPS throughout the investigated period, however, accumulation of cyanotoxins was not detected. In Lake Balaton, on the other hand, cyanobacterial numbers were much lower; more than 400-fold fewer cells/mL were detected during the peak of the bloom. Cyanotoxins were not detected in the water, nor fish tissue samples from Lake Balaton, however, MC- and STX synthetase-coding genes were detected in the filtered biomass, posing a latent danger regarding the possible future blooms in the lake. Lastly, histopathological alterations in the hepatopancreas and kidneys of fish from Lake Balaton were not severe, and more resembled the normal control structure. Therefore, we can assume that the construction of the KBWPS has a significant protective effect, and that it does safeguard Lake Balaton from potentially toxic cyanobacterial blooms. Even though the KBWPS filters most of the inflowing nutrient load from the Zala River, Lake Balaton still faces dispersed inflows of organic waste, and the internal nutrient loads can cause new cyanobacterial blooms. As cyanotoxin-coding genes were detected in the cyanobacterial biomass from Lake Balaton, regular monitoring of this valuable ecosystem for the presence of cyanobacteria and cyanotoxins is of paramount importance, so that the “Hungarian Sea” can remain safe.

## Figures and Tables

**Table 1:** Species, the number of individuals, sex ratio and mean total lengths (mm) of fish sampled from the Kis-Balaton Water Protection System (KBWPS) and Lake Balaton during the investigated period of 2018.

Site	Season	Species	No. Individuals	Sex (Male:Female)	TL (mm)
KBWPS	April	*Cyprinus carpio*	5	2:3	438 ± 62
	May	*Cyprinus carpio*	2	2:0	475 ± 35
		*Carassius gibelio*	5	2:3	384 ± 51
	July	*Carassius gibelio*	5	2:3	355 ± 37
	September	*Abramis brama*	5	2:3	364 ± 42
Lake Balaton	June	*Abramis brama*	5	2:3	234 ± 12
Control	-	*Cyprinus carpio*	3	1:2	355 ± 99

**Table 2:** Primers used for the qualitative PCR for detecting the presence of microcystin (MC; *mcyE*), cylindrospermopsin (CYN; *cyrJ*), saxitoxin (STX; *sxtG*, *sxtS*) and anatoxin (ATX; *anaC*) coding genes.

Gene	Primer Name	5′–3′ Sequence	Reference
*mcyE*	HEPF	TTTGGGGTTAACTTTTTTGGGCATAGTC	[22]
	HEPR	AATTCTTGAGGCTGTAAATCGGGTTT	
*cyrJ*	cyrJ_F	TTCTCTCCTTTCCCTATCTCTTTATC	[23]
	cyrJ_R	GCTACGGTGCTGTACCAAGGGGC	
*sxtG*	sxtG432_F	AATGGCAGATCGCAACCGCTAT	[24]
	sxtG928_R	ACATTCAACCCTGCCCATTCACT	
*sxtS*	sxtS205_F	GGAGTATTDGCGGGTGACTATGA	[25]
	sxtS566_R	GGTGGCTACTTGGTATAACTCGCA	
*anaC*	anaC-genF	TCTGGTATTCAGTCCCCTCTAT	[26]
	anaC-genR	CCCAATAGCCTGTCATCAA	

**Table 3:** Water quality parameters of the Kis-Balaton Water Protection System (KBWPS) and Lake Balaton during the investigated period of 2018.

Physical and Chemical Parameters	KBWPS				Lake Balaton	Guideline ^1^
	April	May	July	September	June	
Temperature (℃)	15.9	24.4	25.5	25.4	23.1	
Conductivity (μS/cm)	726	729	741	757	809	800
pH	9.26	9.42	9.52	9.44	8.54	9
Saturation (%)	87.9	76.1	73.9	41.1	86.1	80
O_2_ (mg/L)	8.53	6.3	5.96	3.38	7.26	7.5
NO_3_–N (mg/L)	<1.0	<1.0	<1.0	<1.0	<1.0	0.06
NO_2_–N (mg/L)	<0.01	<0.01	<0.01	<0.01	<0.01	/
NH_4_–N (mg/L)	0	0	<0.1	1.6	0	0.05
PO_4_–P (mg/L)	1.8	<0.2	<0.2	1.5	<0.2	0.01

^1^ Guideline values are specified by the Hungarian Government Decree [36].

**Table 4:** Qualitative and quantitative composition of the cyanobacterial community of the Kis-Balaton Water Protection System (KBWPS) and Lake Balaton during the investigated period of 2018.

Cyanobacterial Taxon	KBWPS				Balaton
	April	May	July	September	June
*Aphanizomenon flos-aquae* Ralfs ex Bornet and Flahault	115,230	3,936,700	10,975,000	1,968,000	−
*Aphanizomenon hungaricum* Komárková-Legnerová and Mátyás	24,300	53,640	714,500	241,300	−
*Cuspidothrix issatschenkoi* (Usachev) Rajaniemi et al.	14,500	32,100	110,500	74,200	−
*Dolichospermum flos-aquae* (Brébisson ex Bornet and Flahault)	26,000	105,000	214,000	96,300	−
*Dolichospermum spiroides* (Klebhan) Wacklin, L.Hoffmann and Komárek	69,500	1,612,000	956,000	541,000	−
*Microcystis aeruginosa* (Kützing) Kützing	104,300	413,600	525,100	375,000	21,300
*Microcystis flos-aquae* (Wittrock) Kirchner	44,600	332,400	487,000	124,000	−
*Merismopedia glauca* (Ehrenberg) Kützing	+	12,300	9,600	+	−
*Oscillatoria tenuis* C. Agardh ex Gomont	+	+	+	+	10,650
Σ	398,430	6,497,740	13,991,700	3,419,800	31,950

Numbers of cyanobacteria are expressed as number of cells/mL. (+) Present taxa with abundance less than 0.1% of the total number; (−) not present in the sample.

**Table 5:** Presence and concentration of nine MC congeners of the Kis-Balaton Water Protection System (KBWPS) and Lake Balaton during the investigated period of 2018.

MC Variant (µg/L)	KBWPS		Balaton
	July	September	June
MC-LR	1.290	0.316	/
dmMC-LR	/	/	/
MC-RR	0.023	0.015	/
dmMC-RR	/	/	/
MC-YR	0.051	0.009	/
dmMC-YR	/	/	/
MC-LF	0.005	/	/
MC-LY	/	/	/
MC-LW	0.050	/	/

(/) Not detected.

## Data Availability

All relevant data are within the manuscript and its supplementary information.

## Supplementary Materials

The following are available online at https://www.mdpi.com/article/10.3390/microorganisms9050960/s1, Table S1: Classification of the histological alterations in organs analyzed by the semi-quantitative analysis (hepatopancreas, kidney and gills) and their importance factors. Figure S1: Chromatograms and respective UV and MS/MS spectra obtained by HPLC–DAD and LC–MS/MS of microcystin (MC) reference samples (extracts of NIES-107 and PCC7820) and two KBWPS samples (7KBex: July sample; 9KBex: September sample) in which several microcystins were detected as well as a chromatogram and an MS/MS spectrum of cylindrospermopsin (CYN).
